# Monitoring occurrence of SARS-CoV-2 in school populations: A wastewater-based approach

**DOI:** 10.1371/journal.pone.0270168

**Published:** 2022-06-17

**Authors:** Victor Castro-Gutierrez, Francis Hassard, Milan Vu, Rodrigo Leitao, Beata Burczynska, Dirk Wildeboer, Isobel Stanton, Shadi Rahimzadeh, Gianluca Baio, Hemda Garelick, Jan Hofman, Barbara Kasprzyk-Hordern, Rachel Kwiatkowska, Azeem Majeed, Sally Priest, Jasmine Grimsley, Lian Lundy, Andrew C. Singer, Mariachiara Di Cesare

**Affiliations:** 1 Cranfield University, Bedfordshire, United Kingdom; 2 Environmental Pollution Research Center (CICA), University of Costa Rica, Montes de Oca, Costa Rica; 3 Department of Natural Science, School of Science and Technology, Middlesex University, London, United Kingdom; 4 Department of Infectious Disease Epidemiology, Imperial College London, London, United Kingdom; 5 UK Centre for Ecology and Hydrology, Wallingford, United Kingdom; 6 Department of Statistical Science, University College London, London, United Kingdom; 7 Water Innovation & Research Centre, Department of Chemical Engineering, University of Bath, Bath, United Kingdom; 8 Water Innovation & Research Centre, Department of Chemistry, University of Bath, Bath, United Kingdom; 9 School of Population Health Sciences, University of Bristol, Bristol, United Kingdom; 10 Field Services, National Infection Service, Public Health England, London, United Kingdom; 11 Department of Primary Care & Public Health, Imperial College Faculty of Medicine, London, United Kingdom; 12 Joint Biosecurity Centre, Department for Health and Social Care, London, United Kingdom; 13 Institute of Public Health and Wellbeing, University of Essex, Colchester, United Kingdom; University of Helsinki: Helsingin Yliopisto, FINLAND

## Abstract

Clinical testing of children in schools is challenging, with economic implications limiting its frequent use as a monitoring tool of the risks assumed by children and staff during the COVID-19 pandemic. Here, a wastewater-based epidemiology approach has been used to monitor 16 schools (10 primary, 5 secondary and 1 post-16 and further education) in England. A total of 296 samples over 9 weeks have been analysed for N1 and E genes using qPCR methods. Of the samples returned, 47.3% were positive for one or both genes with a detection frequency in line with the respective local community. WBE offers a low cost, non-invasive approach for supplementing clinical testing and can provide longitudinal insights that are impractical with traditional clinical testing.

## Introduction

The role of children and schools in the transmission of SARS-CoV-2 remains a matter of debate [1–3]. Testing data suggested that, at the beginning of the pandemic, children and adolescents (<18 years) account for less than 5% of the overall confirmed COVID-19 cases [4, 5]. However, population surveys have shown a much higher rate of infection in children (11.3% for primary school children and 13% for secondary school children) [6]. This age group is characterised by milder or asymptomatic forms of the disease [7], different symptoms [7] and clinical outcomes from adults [1, 7, 8]. However, children are susceptible to and can transmit SARS-CoV-2 within the school setting and out to the community [9]. Estimates from the REACT-1 study (during the period between the 9 and 27 September 2021) found high and rising infections in school-aged children in England (highest weighted prevalence of swab-positivity in children aged 5 to 12 years was 2.3%, and 13 to 17 years was 2.6%) [10]. Hence, there is a need for better methods to make schools safer environments and more COVID-secure for children and staff. Testing will play a vital role in this [11].

Monitoring SARS-CoV-2 transmission and cases of COVID-19 in schools is challenging. Initial diagnostic testing focused on symptomatic patients, increasing the likelihood that milder cases were missed and no asymptomatic cases were detected [1]. To overcome the limits of symptoms-based testing, mass testing was used to identify positive cases in schools [1]. However, while this approach would provide information on the burden of infection in schools, it is characterised by important organisational (e.g. delivery, parental consent) and economic burdens, questioning its long-term sustainability as a primary surveillance system. Moreover, mass testing provides a picture of the status of the school population at a specific point in time, lending no insight into the days in between testing. Asymptomatic mass-testing in school children using lateral flow devices is not a sustainable long-term approach, with the programme in England ending in February 2022.

Wastewater-based epidemiology (WBE) is a promising non-invasive tool that can support the COVID-19 response as part of an early-warning system, providing data at a local community level proactively to inform public health care strategies and mitigate escalating demands on health care providers [12–14]. SARS-CoV-2 has been identified in adult and child faeces and urine at different stages of the infection [15]. Although most viral shedding which features within wastewater will be derived from faeces. A recent meta-analysis has estimated the mean duration of SARS-CoV-2 shedding in stools to be 17.2 days (95% Confidence Interval 14.4–20.1), longer in duration than in any other body fluid [16]. Another meta-analysis suggested significantly shorter shedding duration in children of 9.9 days (95% Confidence Interval 8.1–12.2) [17] indicating that the window for detection using wastewater in children may be shorter than in the adult population. Wastewater is now widely used as a SARS-CoV-2 surveillance tool at the sewer catchment level via collecting grab and composite samples at wastewater treatment plant inlets [13]. In addition, Near Source Tracking (NST) [18] is conducted at a small sub-catchment scale (i.e., a building), permitting detection of small clusters or even individual COVID-19 cases in locations such as care homes [19], hospitals [20], universities and student dormitories [21, 22], aircraft [12, 23] and cruise ships [12]. Transitions to school environments have not been readily and widely reported to date although illustrations exist for kindergartens [24] and some elementary schools [25] suggesting that WBE / NST may be able to be part of a surveillance system for infectious diseases in schools.

Here, as part of a government-funded epidemiological surveillance pilot program, a wastewater-based epidemiology approach was used to monitor the occurrence of SARS-CoV-2 in wastewater from schools in England. The current paper posits that appropriate frequency monitoring of school wastewater will enable detection of SARS-CoV-2 in a cross-section of education settings (primary, secondary, economic and social strata) and this will be effective as part of a broader public health surveillance system. To test this, wastewater was collected daily across 16 schools within England (UK). Wastewater testing for fragments of SARS-CoV-2 in schools occurred during the initial stages of the pandemic and during the emergence of the Alpha variant of SARS-CoV-2 in Kent, southern England. Here, we report on a case study that explored the WBE approach to identify the presence of SARS-CoV-2 in primary and secondary school wastewater in England (UK).

## Methods

Sixteen schools (10 primary, 5 secondary and 1 post-16 and further education for a total of 17 sites) in England took part in the School wasTEwater-based epidemiological suRveillance systeM for the rapid identification of COVID-19 outbreaks (TERM) study (Table 1). Schools were located across four areas (and neighbourhoods) having different deprivation levels (deprivation was defined based on the quintiles of the Index of Multiple Deprivation a measure of relative deprivation based on a wide range of an individual’s living conditions) [26] and diverse school populations (based on ethnic diversity) and selected among both high and low prevalence areas based on confirmed cases of COVID-19 infection (Table 1). The school population size ranged from 143 to 2061 pupils.

**Table 1:** Schools characteristics.

Phase of education	Frequency of schools
Primary	10
Secondary	5
Post 16	1
**LSOAs quintiles (IMD)**	
Q1—lower	7
Q2	3
Q3	3
Q4	2
Q5 –higher	1
**Pupils classified as white British**	
Very Low: Up to 20%	5
Low: Between 21% and 40%	1
Med: Between 41% and 60%	2
High: Between 61% and 80%	4
Very High: Between 81% and 100%	3
NA	1
	**Min-Max**
**Number of pupils (official)**	143–2061
**COVID-19 cases rate per 100,000 population (cumulative)—October 2020**	209.7–1020.4
**COVID-19 new cases rate per 100,000 population (weekly)—October 2020**	15.1–122.1

Note: IMD: Index of Multiple Deprivation; LSOAs: Lower Layer Super Output Area (community); NA: not available.

### Sample collection

Sampling began on 20th October 2020, approximately six weeks after the start of the 2020–2021 school year. Composite wastewater samples were initially collected twice a week (Tuesday and Thursday; from 8 am to 3 pm) as 7-hour time-proportional composites at a sampling frequency of 60 seconds ‘on’, followed by 4 minutes, ‘off’ using an Aquacell P2-COMPACT (Aquamatic) autosampler. The maximum pumping rate in cases where a steady flow of wastewater was present was approximately 50 ml/min and hence a maximum sample volume of 4.2L was collected. After an initial trial period, a second autosampler (as above) was installed at 7 locations (in 6 schools) to collect samples at a frequency of one minute on/ one minute off between 12 pm and 2 pm (maximum sample volume of 3L collected). The purpose of this more intensive sample collection regime was to evaluate if increasing the frequency of sampling over the lunchtime period (when we assume there is a more significant opportunity to use the bathroom) improved the likelihood of detecting SARS-CoV-2. In addition, from the 4th November 2020, the sampling frequency was increased to 4 days per week (Monday to Thursday). At the end of each school day, one litre from the maximum of 4.2L of wastewater collected in a plastic container was decanted from each autosampler, after thorough mixing, into a separate plastic (polypropylene or polyethylene terephthalate) sample bottle and immediately couriered to the laboratory on melting ice. Sample temperature was checked on receipt in the laboratory. Storage temperatures were monitored daily to ensure a stable temperature in the range of 2.5–4.0°C. Aliquots of these samples underwent RNA extraction (150 ml) and cryogenic sample preservation (200 ml; -80°C) within 24 hours of sample receipt at the laboratory. An overview of sample collection data is provided in Table 2.

**Table 2:** Overview of school locations, sampling dates, collection volumes and ragging (other) incidents that prevented sample collection.

Area (number of schools; the number of autosamplers)	Sample collection dates (start date—end date)	Total number of days of collection	Volume collected	Number of ragging (or other issues^1^) incidents preventing sample collection
Area 1 (4; 6)	20/10/20–17/12/20	83	0–4.2 L	2 (7)
Area 2 (6; 10)	04/11/20–17/12/20	114	0–4.2 L	3 (8)
Area 3 (4; 5)	17/11/20–17/12/20	69	0–4.2 L	0 (1)
Area 4 (3; 3)	10/12/20–17/12/20	14	0.5–4.2 L	0

Key:

^1^ no sample collected for a variety of reasons including ragging (the accumulation of solid wastewater materials greater than 5 mm; number not in brackets) or due to either battery failure, in pipe blockages, autosampler tube out of alignment, unable to access site/inset day or reason not given (summed figure in brackets).

### Protocol for sample analysis

Each wastewater sample was analysed in the laboratory for total suspended solids, ammonium (NH_4_-N), orthophosphate (PO_4_-P), total chemical oxygen demand (tCOD), soluble chemical oxygen demand (sCOD), pH, conductivity, and dissolved oxygen according to standard methods for the examination of water and wastewater [27]. The method used for SARS-CoV-2 RNA analysis is described in Farkas et al. [28]. In detail, school wastewater samples were centrifuged (30 minutes at 3,000 x g at 4°C), and supernatants were spiked with an extraction control murine norovirus before concentration using the polyethylene glycol (PEG) precipitation method with an overnight incubation [29]. A final concentrate was obtained by centrifugation (10,000 × g for 30 min at 4°C), the PEG was removed by pouring, followed by a further centrifugation step / PEG removal step (10,000 × g for 10 min at 4°C) and the resulting pellet resuspended in 0.5 mL of molecular biology grade phosphate-buffered saline (PBS), pH 7.4. Viral extraction from wastewater concentrates was carried out using the NUCLISENS^®^ RNA extraction kit on a MINIMAG^®^ (BioMérieux, France). SARS-CoV-2 RNA detection was performed by RT-qPCR using the RNA UltraSense^™^ One-Step Quantitative RT-PCR System (ThermoFisher, UK) targeting the nucleoprotein (N), N1 fragment and envelope protein (E) gene [30] using a QuantStudio^™^ 7 Pro Real-Time PCR System (ThermoFisher, UK). The RNA extracts were analysed in duplicate alongside negative (nuclease-free water) controls. The RNA extracts were quantified for SARS-CoV-2 titre by plotting the quantification cycles (CT) to an external standard curve constructed from commercially available synthesised plasmids (Integrated DNA Technologies, Leuven, Belgium) containing the target sequence. The empirical limit of quantification (LOD) was calculated based on a method outlined previously [28] and denoted 1270 gene copies per litre (GC / L) for N1 and 3000 GC / L for E. The Limit of Quantification (LOQ) was 9200 GC / L for N1 and 21300 GC / L for E. This was determined through spiking SARS-CoV-2 negative RNA extracts from school wastewater with a range of defined quantities of Armored RNA standard (Asuragen Quant SARS-CoV-2 Panel—52036, VH Bio Ltd., UK), with the LOQ being the lowest concentration which achieved a coefficient of variation (CV) value not exceeding 25%. A positive detection was considered during the study when a sample exceeded LOD alongside no significant amplification in the negative control. This method was validated based on a pilot study involving duplicate extractions from 37 different wastewaters.

For samples which we could positively quantify, the average CV value was calculated (minimum and the maximum CVs given in brackets) for the full protocol which included the concentration, RNA extraction and RT-qPCR steps. For N1 the CV was 9.3% (0.42–23.8) % in wastewaters which ranged from the LOQ to the maximum value of 8 x 106 GC / L (n = 36). The CV for E was 14.5% (0.3–34%) in wastewaters which ranged from LOQ to the maximum value of 9.38 x 106 GC / L (n = 32).

### COVID-19 case rates

Data for the weekly incidence of COVID-19 cases (per 100,00 people) at the community level (defined based on the national geographic hierarchy Middle Layer Super Output Area—MSOA) have been downloaded from the UK Health Security Agency (UKHSA) COVID-19 Dashboard (https://coronavirus.data.gov.uk/details/download). For each school, weekly new cases within the school’s community have been used to summarise community level trends. Given no daily community data were available, for the comparative analysis of wastewater in schools and cases in the community, schools wastewater data were grouped based on the weeks available in the UKHSA database. A lead/lag analysis was performed between the weekly positivity rates in schools and community cases to identify the maximum correlation between the two timeseries using the Pearson’s correlation coefficient.

### Ethics

Ethical approval for this study was obtained from the Middlesex University Ethics Committee (14795).

## Results

**Fig 1:**
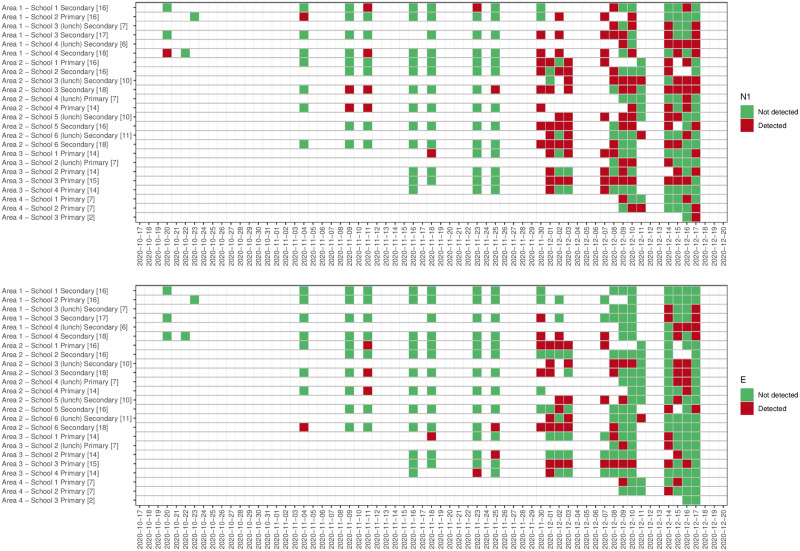
Heatmap detection/non-detection for N1 gene (a) and E gene (b). Note: number of samples collected for each school/sample pattern is provided in square brackets at the end of the name of the school.

**Fig 2:**
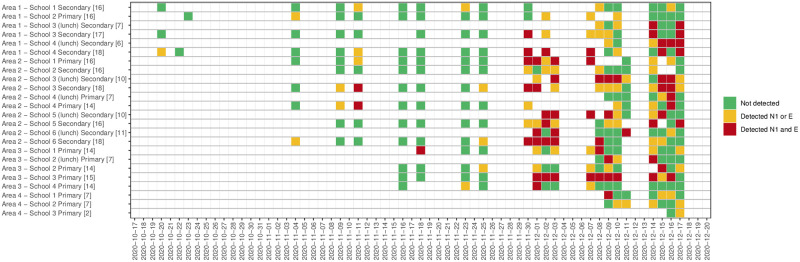
Heatmap detection/non-detection N1 gene and E gene combination. Note: number of samples collected for each school/sample pattern is provided in square brackets at the end of the name of the school.

**Fig 3:**
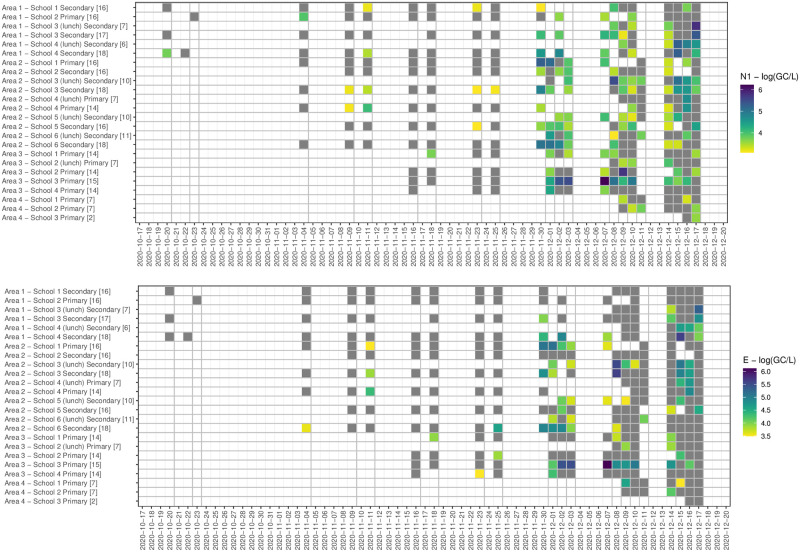
Heatmap daily log10(GC/L) N1 gene and E gene (grey indicated non-detection). Note: number of samples collected for each school/sample pattern is provided in square brackets at the end of the name of the school.

**Fig 4:**
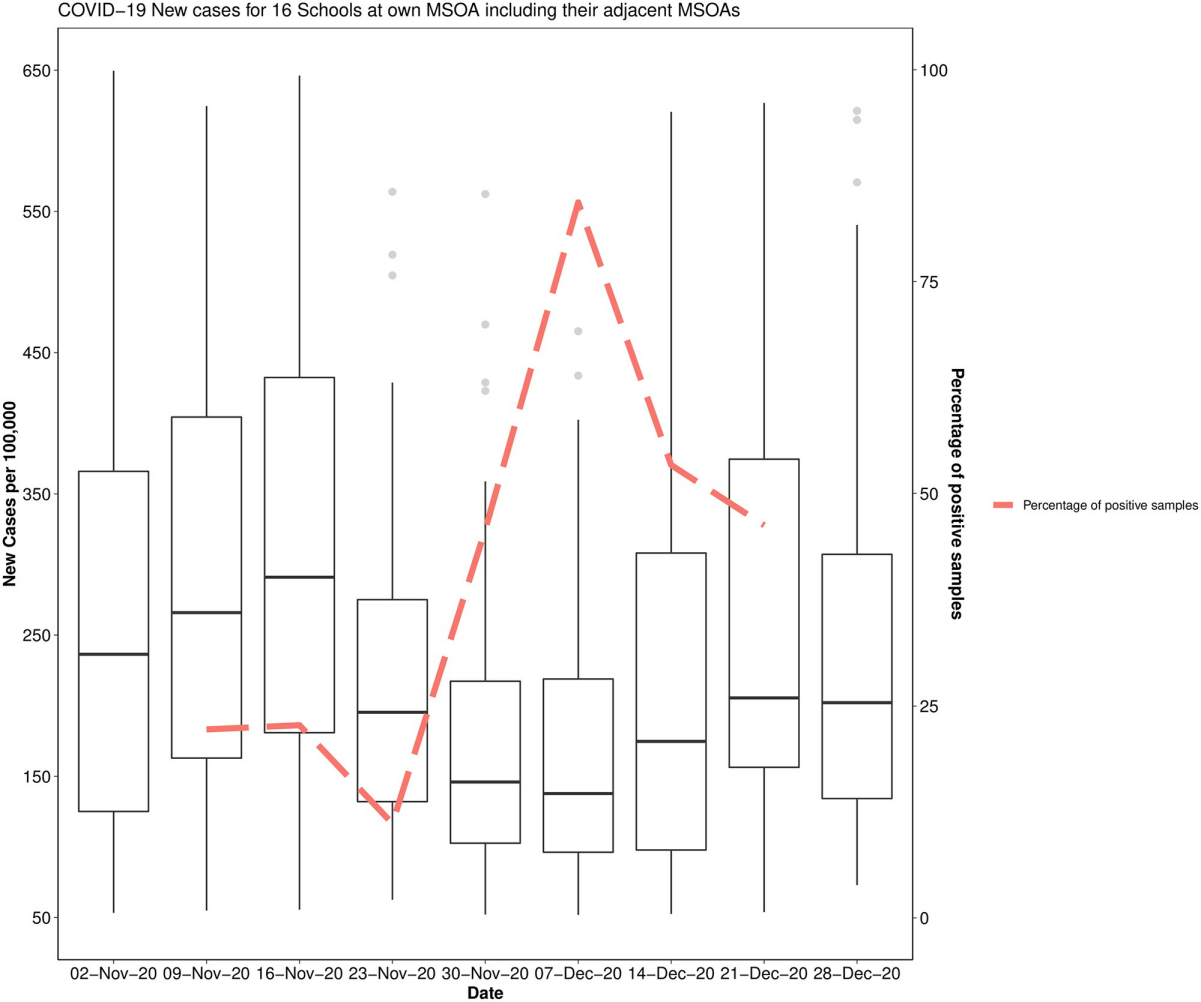
Community level (MSOAs) COVID-19 new cases per 100,000 by week and percentage of positive school samples. Note: Each box shows the distribution of new cases per 100,000 across the community (MSOA*) of each school and all the adjacent communities for a total of 92 MSOAs). Solid lines show medians; the boxes show Interquartile Ranges; the whiskers show ranges. Dashed lines indicate the percentage of weekly positive samples (weeks in this figure are defined based on the MSOA data availability and may differ from the weeks of the study). * MSOAs (Middle Layer Super Output Areas) are a geographic hierarchy designed to improve the reporting of small area statistics in England and Wales. Source: MSOA level data extracted from https://coronavirus.data.gov.uk/details/download.

A total of 296 samples were analysed for eight standard wastewater parameters (including total suspended solids, ammonia and phosphates) and SARS-CoV-2 RNA. In terms of wastewater characteristics, concentrations determined were highly heterogeneous (see **Table 1 in** S1 Appendix for data for all samples and for samples in which the N1 and/or E genes were detected). Median values for all determined wastewater parameters are slightly lower in samples in which a positive signal was detected (see Table 1 in S1 Appendix). However, the reported range for several parameters indicates that SARS-CoV-2 RNA can be detected in school-derived wastewater samples even when, for example, TSS and NH_4_-N concentrations are four orders of magnitude below determined medians (see Table 1 in S1 Appendix), an indication that few individuals have contributed waste products to the sample. 47.3% of the 296 samples collected returned positive for one or both genes. Hundred and twenty-eight (43.2%) samples were positive for the N1 gene, and 75 (25.3%) samples were positive for the E gene. Sixty-three samples were positive for both N1 and E genes (21.3%), 65 samples were positive for the N1 gene but not the E gene (22.2%), and 12 samples (4.0%) were positive for the E gene but not the N1 gene (Figs 1 and 2). In 42.1% of samples, a first positive detection for both genes was pre-dated by a positive detection of one targeted gene and 36.8% by a non-detection (in 21.1% of cases, no sample was collected on the sampling day before the positive case). 80.4% of the samples collected during the first week of December returned positive. This percentage was around 56% and 51% during the second and third weeks of December (Table 3). At the beginning of the study period, the positivity rate in samples collected in primary schools was higher than those observed in secondary schools. From week 5 (last week of November), the positivity rate was consistently higher in secondary school samples than primary schools, with a positive rate of 88.9% in the first week of December. Levels of GC/L varies between 1333 GC/L and 1.68*10^6^ GC/L for the N1 gene target and between 3067 GC/L and 1.31*10^6^ GC/L for the E gene target (Fig 3). The lowest Ct value (highest viral titre quantified) was 31.0 for the N1 gene and 28.2 for the E gene (data not shown). In Fig 4, weekly new COVID-19 cases in the community (reported for each school’s neighbourhood (MSOA) and all the adjacent neighbourhoods, for a total of 92 areas) are presented against the percentage of positive samples (positivity rate) collected each week. Aggregated data suggest an increase/decrease in the detection frequency of targeted genes in line with the increase/decrease in new cases. Specifically, the lead/lag analysis between the weekly positivity rates in schools and community cases shows a maximum correlation between the two-time series when school data are lagged by two weeks (Pearson’s correlation coefficient 0.33, p<0.01), suggesting that the signal in school wastewater precedes the increase in the number of cases in the community. The analysis by area, showed a maximum correlation when school data area lagged by two weeks in area 1 (Pearson’s coefficient 0.63, p<0.01), by 3 weeks in area 2 (Pearson’s coefficient 0.43, p<0.01), and a non-significant correlation in areas 3 (Pearson’s coefficient 0.24, p = 0.25). No specific analysis was performed for schools belonging to area 4 because of lack of sufficient data to define trends in positivity rates (Fig 1 in S1 Appendix).

**Table 3:** Number of samples, percentage of positive cases (total and by stage of education) by week.

	Oct 20–23	Nov 2–6	Nov 9–13	Nov 16–20	Nov 23–27	Nov 30 -Dec 4	Dec 7–11	Dec 14–18
**No. of samples**	5	8	20	25	28	46	75	89
**No. Schools**	4	8	10	14	14	14	16	17
**% positive**	20	25.0	35.0	4	17.8	80.4	56	50.6
**% positive in primary schools (No. of samples)**	0 (1)	33.3 (3)	50.0 (6)	8.3 (12)	14.3 (14)	68.4 (19)	52.9 (34)	43.2 (44)
**% positive in secondary schools (No. of samples)**	25.0 (4)	20.0 (5)	28.6 (14)	0.0 (13)	21.4 (14)	88.9 (27)	58.5 (41)	57.8 (45)

Among both all-day and lunch samples collected in the same school (52 times), 61.5% of these both samples returned consistently positive (or negative); in 23.1% of the occasions, all-day samples were positive (but not the lunchtime) while in 15.4%, the lunchtime samples were positive (but not the all-day).

## Discussion

This study represents a novel application of WBE as it reports on the use of Near Source Tracking (NST) to detect SARS-CoV-2 in primary and secondary schools in England during the mid-stage of the COVID-19 pandemic (i.e. first wave of wild-type) and during the emergence of the SARS-CoV-2 Variant of Concern (VoC) Alpha (B.1.1.7) in the UK. Wastewater detection of the SARS-CoV-2 virus provided compelling evidence that the virus was circulating in schools during the autumn and early winter (median SARS-CoV-2 for N1 5 GC/ml) and that the presence of the virus in the schools was correlated with the community cases with a lag of two weeks. Mass clinical / community testing e.g. Lateral Flow Test or qPCR testing from nasopharyngeal swabs was not routinely available in the UK for the duration of this study. The 16 schools studied here: i) represented a useful subset of the school age population in England, ii) represented diverse communities based on level of economic and social deprivation and school population diversity iii) were selected from four different areas in the country at different stages of community infection levels. The SARS-CoV-2 detection frequency intensified with the increase in new case notifications in the community. Data collected confirm that despite the episodic nature of wastewater flows in schools, the presence of SARS-CoV-2 within the school setting can be identified, which suggests that the use of wastewater epidemiology NST in schools can play a valuable role in monitoring and motivating rapid action. The implications of these findings reinforce the need for continuous monitoring of the wastewater, especially when employing NST with its inherent episodic flow.

An available study [24] illustrates SARS-CoV-2 infections in staff and students in an urban public-school setting via testing wastewater for SARS-CoV-2 alongside weekly saliva samples. Wastewater monitoring for SARS-CoV- 2 RNA was generally consistent with the detection of SARS-CoV-2 infections by saliva testing although at some points negative results were against the positive saliva test results. A preprint by Fielding-Miller et al. [25] demonstrated 29.8% positive rate of SARS-CoV-2 in wastewater samples from elementary schools in San Diego, USA in the academic year of 2020/2021. This is lower than the positivity rate found in this study (47.3% for one or both genes over the total sample period). In addition, they determined an 83.7% positive detection rate in surface water samples which is similar to our detection rate in the first week of December (88.9%) [25].

A further publication by Gibas et al. investigated the presence of SARS-CoV-2 in wastewater from student halls of residence at the University of North Carolina at Charlotte from September to November 2020. Here, they found a significantly lower rate of positivity than this study (47.3% for one or both genes) with only 12% of true positive samples and 4.5% of suspected positive samples [22].

Viral material detected in samples has three possible origins: shedding from students and/or staff and/or visitors. Validating the source of viral shedding requires regular asymptomatic testing of students and staff; during the analysis period, this was not the strategy which was performed in English schools. Clearly, having the longitudinal sampling benefit of cheap and non-invasive WBE alongside the option of individual clinical testing would offer insights into the circulation of the virus within school settings. This complementary approach was pioneered in a study in Arizona State for University students [31, 32] and later in schools [25]. The study by Fielding-Miller et al. highlighted that WBE increased the acceptance of routine and targeted clinical testing, by providing data led incentives and social acceptance in educational settings which are typically sites of low vaccine availability / acceptance and have hesitancy for other forms of testing. The study successfully identified 93% of on-campus COVID-19 cases with a combination of wastewater and surface monitoring for SARS-CoV-2 [25]. Genomic sequencing of SARS-CoV-2 VoC in NST applications provides an opportunity to monitor for potentially more virulent or transmissible variants or perhaps emergence of new diseases. WBE in NST applications including schools provides an efficient means of informing policy decisions for public health control.

Few, if any, data are available on the toilet use habits of either primary or secondary school-aged children in the school setting. The data provide circumstantial evidence that sufficient school attendees contribute to the waste stream, although the authors acknowledge that a small number of infected individuals (especially adults / staff / visitors) could provide a significant and detectable viral signal. However, we argue that the transmission of COVID-19 is well established in children and that faecal shedding alongside nasopharyngeal shedding is expected in this group. In this study >52% of samples were positive SARS-CoV-2 throughout the study. Evidence supporting loose bowel movements is emerging in the literature [33], with 19% of children who tested positive reporting gastrointestinal symptoms, including diarrhoea [34]. However, it remains unknown the extent to which the frequency of bowel movements increases in mild or asymptomatic children with COVID-19. SARS-CoV-2 in urine, vomit, handwashing and/or mucus (from nose-blowing) cannot be ruled out as a source of some positive detects in the wastewater. Based on this limited pilot data, it is recommended that all day sampling is undertaken for wastewater in schools. This maximises the collection period which is vital for detection of transient events.

Surveillance data from NST were used to confirm the presence or absence of SARS-CoV-2 in the wastewater. Although the data are quantitative, it is not currently possible to identify the number of unique flush events represented within a composite sample. Therefore, it is tempting to normalise the data to the population, for example, to estimate the proportion of the sample population infected. Still, the number of individuals who have had a bowel movement on a given day remains unknown and this data in school age children and adolescents is scant, subject to significant uncertainty and a high degree of variability. The case for disruptive public health interventions in educational settings remains controversial. We propose on the basis of this study that a theoretical public health intervention (e.g. clinical testing and isolation) should not be dependent on the number of infected people, with mass testing performed on first detection (e.g. SARS GC >LOQ) to identify infected individuals and support their isolation to prevent classroom or school closure which are more disruptive to the student body as a whole. Given the uncertainty around translating wastewater concentrations of SARS-CoV-2 into counts of infected individuals, NST is, at this stage, can be employed as a surveillance tool, by signalling the presence of the virus, rather than as a measure of community transmission. In addition, collection of data more aligned across a fine geographical resolution would even allow for methodological advancements and analyses able to estimate the actual causal links. However, further work (i.e. introduction of continuous sampling, a better understanding of viral shedding and robust modelling approaches) is required to estimate the number of infected individuals. We suggest that positive wastewater detection should promote less disruptive public health measures in educational settings. Fielding-Miller et al. suggested that risk mitigation measures such as masking/double-masking, social distancing, and ventilation could be an appropriate intervention [25]. It is proposed here that this could be used alongside traditional public health measures (i.e. isolation, household testing and online learning provision) prior to return to education while waiting for responsive testing implementation and results, and/or in the absence of identifying a case.

There is still some uncertainty regarding the utility of qualitative measurements (≥LOD) vs quantitative measures (≥LOQ), considering uncertainties resulting from the sampling and analysis. As a surveillance tool, the primary function is to find a pathogen in locations that it should not be, i.e., a school. Hence, using the LOD (presence/absence) is perhaps justified as it demonstrates the main analyte is present in the sampled population. However, a school sample that reports <LOD is still at risk of false negatives given the limitations of the autosampler and its sampling schedule linked with the probability of capturing stool from all infected individuals (i.e. even a composite sample is only a fraction of the flow over a given period). To illustrate this issue, the autosampler used in this study is time-weighted, collecting a sample at a predefined time period which is independent of flow. If the sample collection time period coincides with a no flush or post-flush period the faeces of an infected individual may be missed. In this scenario, one might expect either no or deficient levels of SARS-CoV-2 in the autosampler reservoir, yielding a detectable (i.e. >LOD) but not quantifiable (<LOQ) result. In particular, if both the probability of toilet uses in school and the probability of an individual in the school being infected are considered together with the likelihood of the autosampler to capture stool from an infected individual (assuming that only 20% of the flow is sub-sampled), it is evident that the possibility of a positive detection is extremely low. To illustrate: an optimistic scenario of 50% of flushes containing faecal material, 1% positive cases in the school population, and 20% of wastewater sampled yields a probability of detecting an infected individual’s stool at <0.1%. Yet, we report the detection in approximately 50% of the samples. The apparent deviation from our assumptions suggests that additional research is needed to explore toilet behaviours in schools, faecal shedding among asymptomatic/pre-symptomatic individuals, and prevalence of gastrointestinal symptoms among infected young individuals.

The focus of future sampler innovation should be the need for a more efficient means of obtaining representative samples (e.g. flush driven sample collection) by a readily available, cost effective and straightforward means if effective virus monitoring is to be implemented in schools.

While this study has provided evidence of the use of WBE in school settings, there is very limited information on the best practices and specific guidance on how local health protection teams can respond to NST WBE data. An approach is needed to co-produce with stakeholders’ protocols to integrate these data into existing practices which will define when, how, and to whom the data should be shared. However, the public health benefits must be constantly weighed against ethical trade-offs since human waste products contain a wealth of information about behaviours and health status. At the level of a single building, it is not easy to preserve donor anonymity. As well as potentially infringing on individual autonomy, this raises the risk of stigmatising businesses, communities, or individuals with associated financial and social disbenefits. NST methods and tools are developed to support ethical sampling strategies and robust and transparent reporting to inform public health action [35, 36].

Other limitations of NST involve the practicality of sampling. In WBE that is undertaken at the wastewater treatment plant immediately before treatment, homogenous, large volume samples can often be obtained to give representative data on the study population. NST, where sampling occurs in the pipe exiting the building in question, can result in low flow events, lack of homogenisation of sewage and missed flush events (where the autosampler schedule is off during a flush of an infected individual) [37]. In addition, as can be seen in Table 2, we had a number of instances where ragging or pipe blockages were an issue meaning that composite samples were not collected.

Despite its limitations, the data support the use of wastewater epidemiology NST in schools as a tool to trigger outbreak investigation or asymptomatic testing. This approach could be used to track outbreaks (evidenced by log increase in GC value above LOD in new outbreaks), or several log changes in baseline SARS-CoV-2 in schools with existing low-level detection (i.e. after an outbreak has receded). Further work is needed to identify suitable triggers of threshold values for each of these case studies. It is important to note, however, that any detection of SARS-CoV-2 in a school environment should be considered as potentially important. To enable the long-term sustainability of WBE to enhance resilience in public health response in schools and other vulnerable or under-sampled populations. It is acknowledged that monitoring schools at an appropriate frequency and processing of samples to enable suitable time to detection is needed for WBE to act as an early warning system. Communication of positive signals within a school community represents the opportunity to develop a targeted approach. Superior knowledge of the presence of SARS-CoV-2 in a school community permits more robust communication messaging about the need for mass testing of individuals and can help overcome barriers of risk denial/displacement and the commonly observed testing hesitancy. Results from the first round of the Schools Infection Survey (ONS) suggest that the participation rate among pupils was on average 17% (51% among staff) [38]; highlighting a critical need to encourage the increased uptake of mass testing amongst school pupils their families. In addition, regular feedback of results to each school and its wider community may improve engagement with non-pharmaceutical interventions and implementation of infection prevention and control measures such as decreasing bubble sizes, staggering start/ finish/ break times and enhanced cleaning protocols. Finally, WBE could provide valuable insights on the local epidemiology of COVID-19 into areas/schools with low uptake of school mass testing and a high level of hesitancy to vaccination.

## Supporting information

S1 Appendix (DOCX)

S1 Data (PDF)
