# Supplementary material for: Monitoring occurrence of SARS-CoV-2 in school populations: A wastewater-based approach
Source: PLoS One. 2022 Jun 17;17(6):e0270168. doi: 10.1371/journal.pone.0270168 (PMC9205509; doi:10.1371/journal.pone.0270168)

**Appendix**

Table 1. Overview of crucial wastewater characteristics in samples collected from all schools and in samples where the N1 and/or E gene were detected*

|  | **N** | **Minimum** | **Maximum** | **Median** | |
| --- | --- | --- | --- | --- | --- |
| **pH** | 248 (108) | 6.0 (6.1) | 9.8 (9.5) | | 7.8 (7.5) |
| **Conductivity (µS)** | 248 (108) | 57 (57) | 5734 (5086) | | 1687 (1537) |
| **Dissolved Oxygen (mg/L)** | 248 (108) | 0.1 (0.2) | 12.1 (11.8) | | 3.0 (2.8) |
| **Total Suspended Solids (mg/L)** | 248 (108) | 0.1 (0.7) | 9844 (9844) | | 150 (140) |
| **NH4-N (mg/L)** | 248 (108) | 0.6 (0.6) | 113.9 (113.9) | | 44.4 (39.5) |
| **PO4-P (mg/L)** | 248 (108) | 0.3 (0.3) | 72.5 (25.6) | | 4.6 (4.6) |
| **Total COD (mg/L)** | 243 (106) | 10 (23) | 3512 (3512) | | 333 (295) |
| **Soluble COD (mg/L)** | 247 (108) | 5 (5) | 1692 (1268) | | 85 (79) |

Key: * = numbers in brackets refer to samples in which a positive signal was detected; n = number of samples

Figure 1 - Community level (MSOAs) COVID-19 new cases per 100,000 by week and percentage of positive school samples across four areas (Red line shows the new cases rate at community level and black dashed line shows the positivity rate of wastewater samples). Note: Loess regression used to estimate solid lines.


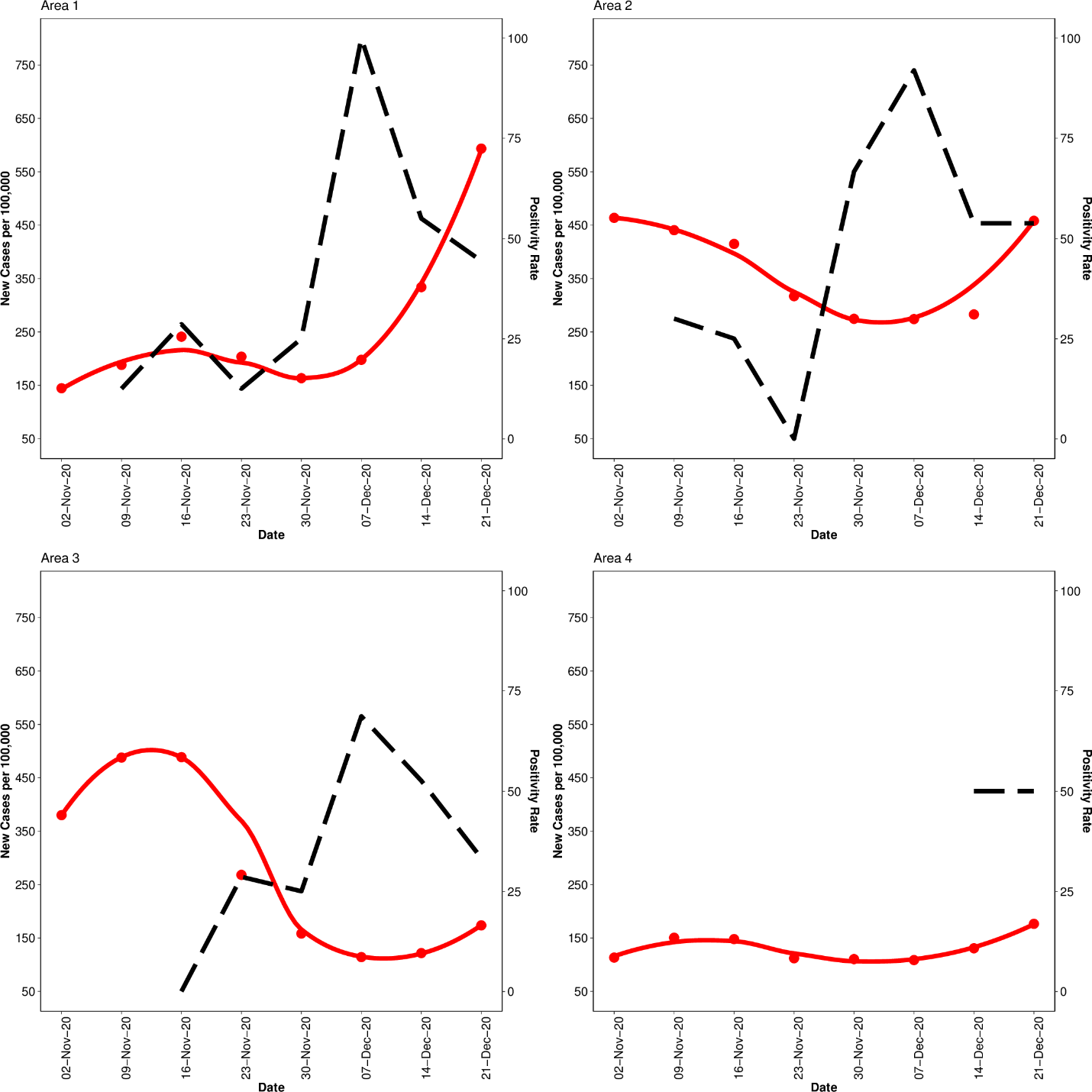

Supplement: S1 Appendix — (DOCX) [file pone.0270168.s001.docx]
