# Supplementary material for: Monitoring occurrence of SARS-CoV-2 in school populations: A wastewater-based approach
Source: PLoS One. 2022 Jun 17;17(6):e0270168. doi: 10.1371/journal.pone.0270168 (PMC9205509; doi:10.1371/journal.pone.0270168)
Supplement: S1 Data — (PDF) [file pone.0270168.s002.pdf]

Date	CtN1	GC/ml-N1	CtE	GC/ml-E	pH	Conductivity	Dissolved-Oxygen	Total-Suspended-Solids	NH4	PO4	tCOD	sCOD
20/10/2020	37.20	5.60	40.14	1.20	7.4	811	6.53	16	24.5	1.6	294	76
20/10/2020					7.56	567	9.64	46	4.4	2.3	342	70
20/10/2020					7.53	1097	5.1	687	57.8	4.1	NA	NA
22/10/2020					7.74	444	9.96	4	2.6	0.6	489	428
23/10/2020					8.59	1225	3.45	258	88.3	2.5	515	355
04/11/2020					8.25	734	9.08	31	36.8	2.5	272	45
04/11/2020	42.57	0.13	39.65	0.27	8.43	980	8.06	84	56.5	3.5	35	25
04/11/2020	41.91	0.13			6.91	307	8.41	514	3.7	3.2	148	53
04/11/2020					7.3	726	7.52	201	28.4	3.8	304	80
04/11/2020	35.62	8.67	39.19	0.27	8.36	854	5.11	76	48.2	2.5	274	64
04/11/2020			40.24	0.13	6.74	249	7.54	79	13.5	2	375	90
04/11/2020					7.12	608	6.41	62	5	1.9	746	363
04/11/2020			35.14	3.73	6.54	57	11.81	3	1.5	21	61	25
09/11/2020					7.65	1156	1.34	321	80	21	766	330
09/11/2020	41.68	0.13			8.05	744	7.23	98	31.3	1.9	167	49
09/11/2020					7.63	366	1.92	146	36.1	3.8	221	146
09/11/2020			43.66	0.00	7.97	800	3.32	263	36.2	3.2	264	193
09/11/2020	38.41	1.47	36.63	1.47	7.02	252	8.31	67	6.4	2.5	93	25
09/11/2020					7.29	320	0.7	898	22.8	11	1500	598
09/11/2020					8.63	759	2.78	280	32.1	2.5	364	242
09/11/2020	38.47	1.33	38.57	0.40	7.29	320	0.7	898	22.8	11	1500	598
09/11/2020					7.77	325	4.02	278	13.6	3.5	549	285
09/11/2020					7.52	531	9.78	26	2.2	2	25	25
11/11/2020	37.86	12.00	35.42	21.73	6.14	451	6.39	758	33.4	18	1578	351
11/11/2020	41.24	1.47			7.4	463	8.7	27	2.1	2.1	25	25
11/11/2020					9.24	1685	1.03	112	80	23	432	255

11/11/2020	40.41	2.53	37.27	7.47	7.07	521	6.64	42	24.4	3.6	25	25
11/11/2020					8.62	777	2.23	307	54.1	2.1	352	25
11/11/2020					9.79	1785	2.58	1760	80	20	1783	588
11/11/2020	40.28	2.80			7.17	589	6.78	100	14.7	1.4	33	25
11/11/2020	42.53	0.93	38.70	3.07	7.16	364	3.33	331	21.9	4.5	392	25
11/11/2020					6.18	374	0.72	81	19.8	1.8	198	25
11/11/2020					8.14	795	0.9	102	48.9	4.2	142	25
16/11/2020					7.67	1069	10.95	12	5.5	1.1	25	44
16/11/2020	42.97	0.53			8.62	1650	8.67	224	25.5	0.5	316	35
16/11/2020					9.45	3776	1.18	223	80	13	913	207
16/11/2020					6.82	2082	0.84	1560	77.8	57	1786	757
16/11/2020					6.8	703	2.38	132	0.7	0.5	239	25
16/11/2020					6.96	1505	0.62	1820	51.9	46	1822	363
16/11/2020					6.84	655	2.47	36	3.5	0.8	102	10
16/11/2020					6.65	638	2.53	301	1.3	1.8	531	71
16/11/2020					7.66	1388	9.15	52	4	0.8	56	25
16/11/2020					7.45	1798	1.84	65	33.7	0.8	283	167
16/11/2020					9.11	2470	2.44	119	80	4.6	172	25
16/11/2020					9.51	3497	1.17	182	80	4.2	275	67
18/11/2020					8.31	1950	5.02	128	25.1	3.9	254	23
18/11/2020	39.18	5.20	37.05	8.40	8.83	1726	4.31	56	45.1	2.7	84	25
18/11/2020					7.56	3555	0.42	2440	80	73	2435	682
18/11/2020					9.17	2098	4.26	33	47.1	1.6	191	36
18/11/2020	42.71	0.53			9.12	1976	5.18	104	40.8	0.5	190	42
18/11/2020	43.93	0.27			9.06	959	9.42	10	39.3	2.9	25	25
18/11/2020					8.67	1749	1.25	90	56.1	4	156	36
18/11/2020	43.76	0.27			6.18	2317	1.02	3180	99.2	73	2960	1547
18/11/2020					8.02	1585	7.25	18	17.9	0.5	47	25
18/11/2020	43.13	0.40			6.85	2067	1.55	214	41.5	5.4	835	364
18/11/2020					7.48	956	5.64	2999	13.8	2.3	151	25

18/11/2020	43.78	0.27			6.35	817	1.1	5800	12.6	1.6	370	108
18/11/2020	43.16	0.40			7.9	1610	8.83	142	6.41	0.5	25	25
23/11/2020	42.87	0.80			7.27	1477	6.62	35	6.8	14	48	57
23/11/2020	45.59	0.13			8.33	2309	2.65	35	75.5	7.9	257	65
23/11/2020					8.99	2391	0.66	202	110	11	594	198
23/11/2020	42.76	0.93			7.74	2281	0.06	1076	46	11	1753	291
23/11/2020	42.31	1.20			7.75	2191	0.44	92	60	5.4	262	101
23/11/2020	44.09	0.40	39.72	3.07	9.08	3607	3.07	1432	80	6.1	1735	232
23/11/2020					7.68	1175	8.41	10	4	0.4	96	121
23/11/2020	42.35	1.20			6.83	863	1.5	77	16.8	3	237	90
23/11/2020					8.33	1997	0.38	1140	37.3	9.9	1035	34
23/11/2020					7.99	2411	0.81	33	80	9.1	180	128
23/11/2020					6.9	826	0.53	509	7.8	4.4	1003	104
23/11/2020					8.5	3637	0.75	2060	80	23	2290	609
23/11/2020	42.68	0.93			8.33	2309	2.65	99	75.5	7.9	257	135
23/11/2020	41.93	1.47			7.49	1558	3.93	284	15.3	3.6	450	116
25/11/2020					7.11	1911	0.26	558	30.6	4.7	1042	232
25/11/2020					8.89	2568	4.93	588	93.4	5.2	988	93
25/11/2020					NA	NA	NA	NA	NA	NA	NA	NA
25/11/2020			38.28	7.47	8.08	1953	1.12	597	40.4	5.4	754	78
25/11/2020					7.46	1510	0.6	2728	11.2	8.7	1907	320
25/11/2020	44.64	0.27			8.74	1860	3.06	164	20.9	1.7	335	63
25/11/2020	43.02	0.80	40.51	1.87	8.12	2038	5.02	58	24	1.8	165	68
25/11/2020	43.59	0.53			8.63	2171	1.85	461	43.1	3.2	589	57
25/11/2020			41.33	1.20	7.99	605	5.84	181	13.3	2.7	347	31
25/11/2020					6.68	2695	0.44	7760	80	25	2765	1692
25/11/2020	42.21	1.33	41.57	0.93	8.08	1770	1.3	77	47	4.8	244	103
25/11/2020					8.48	1935	1.61	3940	77.1	11	1921	213
25/11/2020			41.91	1.33	7.4	497	8.07	22	5.7	1.6	44	44
25/11/2020			35.63	39.60	9.39	2814	0.95	46	80	10	238	135

30/11/2020	37.96	35.47	34.61	24.80	8.51	1103	1.33	75	71.6	3.1	110	39
30/11/2020	39.82	12.27	36.13	9.47	7.82	936	0.64	964	54.7	4.6	1670	62
30/11/2020	43.56	1.20			7.27	499	3.64	77	1.7	2.1	128	12
30/11/2020	36.13	107.60	32.57	86.00	8.81	850	1.07	178	94.4	6.7	285	108
30/11/2020	42.00	3.33			6.35	1045	0.47	7528	85.1	25	3512	1144
30/11/2020	35.17	191.73	31.98	126.13	9.21	1397	2.68	207	80	8.6	405	74
30/11/2020	41.57	4.00			6.33	143	4.14	111	3.6	0.6	117	5
30/11/2020	36.86	69.20	33.13	63.87	7.22	251	2.84	88	20.3	2	142	13
30/11/2020	41.99	3.07			6.96	719	0.22	6240	75.1	13	3380	151
30/11/2020					8.6	765	2.59	96	27.3	1.8	167	44
01/12/2020	41.47	4.27			9.17	1427	1.57	2142	80	6	1801	176
01/12/2020	38.81	23.20	35.34	15.07	6.65	736	0.77	517	34.5	3.5	875	142
01/12/2020	41.20	5.07			8.91	1304	1.14	888	80	6.8	1036	111
01/12/2020	36.02	114.53	32.89	70.40	NA	NA	NA	NA	NA	NA	NA	NA
01/12/2020	38.11	32.67	36.79	6.00	NA	NA	NA	NA	NA	NA	NA	NA
01/12/2020					9.26	784	1.14	78	80	8.9	290	66
01/12/2020	35.64	143.47	31.87	132.93	7.07	383	1.59	85	22.6	2.9	124	21
01/12/2020	40.39	8.27			8.68	860	3.81	408	80	9.6	815	93
01/12/2020			35.93	11.47	7.29	278	7.43	504	7.4	3.1	226	6
01/12/2020	40.90	6.67	36.68	6.40	8.7	773	0.62	1980	114	16	1732	183
01/12/2020	38.69	22.93	35.00	19.07	7.45	583	4.1	34	18.1	3.4	191	62
02/12/2020	41.61	4.00			7.68	670	2.22	1184	25.9	4.2	2374	125
02/12/2020	37.18	57.47	32.68	81.20	7.81	688	4.42	57	30.6	2.6	204	45
02/12/2020	40.57	7.33			7.67	648	1.98	328	25.4	3.5	101	59
02/12/2020					8.43	680	5.73	226	50	5.2	564	81
02/12/2020					8.94	930	2.53	569	80	5.8	822	149
02/12/2020	35.02	251.47	30.70	279.20	6.89	562	0.94	150	22.3	3.2	295	178
02/12/2020					8.97	1070	2.29	563	80	5.2	869	117
02/12/2020			35.07	17.87	7.41	297	7.74	421	9.2	3.2	529	28
02/12/2020	41.85	4.13			8.88	988	3.34	508	80	14	1013	272

02/12/2020					9.38	1505	1.63	73	80	17	285	155
02/12/2020	37.19	64.27	32.65	81.87	9.23	1263	1.91	1596	80	10	1866	187
02/12/2020	41.73	3.60	35.70	12.00	6.71	467	1.55	203	37	2.6	422	169
02/12/2020	40.17	10.13	35.69	12.93	6.73	288	2.23	40	20.1	1.3	190	56
03/12/2020					NA	NA	NA	NA	NA	NA	NA	NA
03/12/2020	42.19	2.80			NA	NA	NA	NA	NA	NA	NA	NA
03/12/2020					NA	NA	NA	NA	NA	NA	NA	NA
03/12/2020	34.35	314.13	31.51	306.67	NA	NA	NA	NA	NA	NA	NA	NA
03/12/2020	43.28	2.93			NA	NA	NA	NA	NA	NA	NA	NA
03/12/2020	42.50	4.53	36.30	4.53	NA	NA	NA	NA	NA	NA	NA	NA
03/12/2020	41.27	9.07			NA	NA	NA	NA	NA	NA	NA	NA
03/12/2020	41.62	8.27	35.47	8.00	NA	NA	NA	NA	NA	NA	NA	NA
03/12/2020	41.24	11.07	36.19	4.80	NA	NA	NA	NA	NA	NA	NA	NA
03/12/2020	42.91	3.60			NA	NA	NA	NA	NA	NA	NA	NA
03/12/2020	42.22	6.00	35.27	9.20	NA	NA	NA	NA	NA	NA	NA	NA
03/12/2020	41.15	9.73	36.34	4.40	NA	NA	NA	NA	NA	NA	NA	NA
07/12/2020	43.57	2.53			NA	NA	NA	NA	NA	NA	NA	NA
07/12/2020	42.58	4.53	38.84	0.80	NA	NA	NA	NA	NA	NA	NA	NA
07/12/2020	32.10	1683.87	28.18	1313.07	NA	NA	NA	NA	NA	NA	NA	NA
07/12/2020	42.31	5.07			NA	NA	NA	NA	NA	NA	NA	NA
07/12/2020	40.35	15.33			NA	NA	NA	NA	NA	NA	NA	NA
07/12/2020	43.15	3.07			NA	NA	NA	NA	NA	NA	NA	NA
07/12/2020	41.77	6.93	35.60	7.33	NA	NA	NA	NA	NA	NA	NA	NA
07/12/2020	41.04	10.40	36.51	4.00	NA	NA	NA	NA	NA	NA	NA	NA
07/12/2020	41.07	11.07	36.53	3.87	NA	NA	NA	NA	NA	NA	NA	NA
08/12/2020	41.33	10.27			7.84	1997	1.51	60	32.8	3.5	65	31
08/12/2020	40.71	12.93			7.44	2426	0.49	2052	49.9	10	447	185
08/12/2020	41.90	6.40			7.07	5086	0.17	9844	80	26	1873	1268
08/12/2020					7.13	1809	1.76	426	29.1	3.5	188	47
08/12/2020					8.96	2533	5.77	107	90.1	4.3	171	38

08/12/2020					8.85	2838	2.89	529	80	6.2	821	87
08/12/2020	36.90	110.27	32.35	73.07	6.52	2475	0.17	1496	57.8	13	449	586
08/12/2020	42.61	4.27	36.07	5.20	8.79	3492	0.41	680	80	5.4	808	149
08/12/2020	35.07	312.40	30.03	345.47	7.08	2457	7.63	17	15.3	1.8	51	21
08/12/2020					6.94	735	4.57	26	15.1	1.5	110	43
08/12/2020			29.77	406.80	6.92	2372	5.69	83	15.6	1.8	106	26
08/12/2020	42.77	2.53			8.31	2836	2.59	1028	80	13	1140	106
08/12/2020	44.74	1.20			9.3	3582	3.41	49	80	13	83	63
08/12/2020	40.60	10.40	36.42	4.13	9.12	2896	0.75	614	80	9.4	424	108
09/12/2020	42.65	2.67	36.54	38.67	9.17	3068	1.21	129	80	11	122	62
09/12/2020					8.25	1222	4.08	19	44.7	4	70	24
09/12/2020	34.22	559.33			6.98	2094	0.25	368	48.6	5.7	663	56
09/12/2020	42.54	2.80	38.23	8.13	9.15	2838	0.55	85	114	3.2	85	73
09/12/2020	44.64	0.80			9.1	3069	0.73	467	80	4	740	200
09/12/2020	39.98	14.53	34.28	55.20	6.47	2713	0.31	3264	68.3	16	1786	923
09/12/2020					8.91	3938	0.54	348	80	5.4	429	111
09/12/2020	42.43	4.67			7.35	2170	4.22	207	32.5	1.2	283	64
09/12/2020					7.69	1331	5.82	2	1.8	1.6	10	10
09/12/2020					7.09	3455	0.38	2236	91.2	20	2093	586
09/12/2020	43.61	1.47			7.34	2178	2.51	852	42.8	6	1193	169
09/12/2020					7.83	2005	5.82	26	25.7	1.2	45	19
09/12/2020					9.11	2161	0.22	61	97.4	8.4	118	62
09/12/2020	42.77	2.80	40.42	3.33	6.48	852	0.45	147	2.9	0.9	109	26
09/12/2020	41.30	6.40	35.87	20.53	7.35	1911	0.83	133	46.2	4.5	118	56
09/12/2020	42.20	3.87			6.76	856	0.3	272	3.5	1.3	235	73
09/12/2020	41.06	7.73	45.80	0.13	7.66	1888	0.73	68	44.4	4.3	47	41
09/12/2020					9.08	2352	0.68	102	112	8	100	68
09/12/2020					6.85	4978	0.2	528	80	23	1846	1008
09/12/2020			45.71	0.13	6.02	4896	0.25	299	80	22	891	473
10/12/2020	40.83	2.13			7.73	2715	3.03	0.94	59.2	2.7	363	56

10/12/2020					7.48	2443	2.34	0.78	44.3	2.3	166	57
10/12/2020					7.59	2419	0.36	3.59	51.5	5.6	879	84
10/12/2020	40.47	2.67			8.68	4054	0.28	5.78	80	11	1426	168
10/12/2020					7.84	1358	9.4	0.06	2.7	1.9	13	6
10/12/2020					9.45	5734	1.56	0.79	80	23	194	85
10/12/2020	40.77	2.27			NA	NA	NA	NA	NA	NA	NA	NA
10/12/2020	40.86	4.00			8.55	2576	8.54	63	90.2	4.8	64	39
10/12/2020					7.13	1824	3.07	274	34.9	6.1	306	57
10/12/2020					7.73	2732	7.41	173	88.1	5.7	321	56
10/12/2020	35.46	90.13	36.05	113.87	7.37	3377	1.05	2496	80	25	2875	664
10/12/2020					8.7	4759	2.04	571	80	11	597	92
10/12/2020	39.77	4.53			7.72	2260	1.14	10.43	38.5	6.7	1256	67
10/12/2020	39.82	4.00			NA	NA	NA	NA	NA	NA	NA	NA
10/12/2020					9.23	3408	2.48	0.78	80	9.9	134	37
10/12/2020					9.15	3548	0.88	0.82	80	9.8	153	38
10/12/2020					7.05	1667	0.8	5.62	75.6	16	1062	174
10/12/2020	38.09	12.27			7.69	1697	0.42	0.73	45.4	3.1	204	107
10/12/2020	41.33	1.60			6.84	780	7.26	0.86	6.4	2.4	351	42
10/12/2020	41.03	1.87			7.38	1404	5.47	1.54	28.3	4	340	73
10/12/2020	39.83	4.00	40.11	4.13	7.14	1430	5.98	0.69	32.4	4.5	129	56
10/12/2020					8.9	2603	1.04	6.83	80	11	635	55
11/12/2020					NA	NA	NA	NA	NA	NA	NA	NA
11/12/2020					NA	NA	NA	NA	NA	NA	NA	NA
11/12/2020					NA	NA	NA	NA	NA	NA	NA	NA
11/12/2020	41.29	6.00			NA	NA	NA	NA	NA	NA	NA	NA
11/12/2020					NA	NA	NA	NA	NA	NA	NA	NA
11/12/2020					NA	NA	NA	NA	NA	NA	NA	NA
11/12/2020					NA	NA	NA	NA	NA	NA	NA	NA
11/12/2020	41.33	5.73	39.60	11.20	NA	NA	NA	NA	NA	NA	NA	NA
11/12/2020					NA	NA	NA	NA	NA	NA	NA	NA

11/12/2020	41.34	5.73			NA	NA	NA	NA	NA	NA	NA	NA
14/12/2020					8.22	1126	10.36	11	4.6	0.9	21	20
14/12/2020	40.85	2.13			7.62	1127	10.33	16	4.5	0.8	23	20
14/12/2020	40.91	2.00			8.63	3673	2.09	97	80	12	187	106
14/12/2020					7.03	5171	0.95	1131	80	14	1802	651
14/12/2020	40.67	2.40	39.80	14.13	8.04	2015	7.39	54	29.9	3.2	109	57
14/12/2020	39.39	5.47	41.41	6.00	7.91	3636	1.51	300	80	5.8	844	116
14/12/2020	40.99	1.87			7.28	1689	4.14	212	69.4	13	493	149
14/12/2020					7.2	1826	1.28	11	21.6	1.3	366	85
14/12/2020	38.33	11.33	37.26	58.00	6.45	2288	2.06	1084	58.1	11	1885	313
14/12/2020			40.57	9.20	8.41	2899	7.92	127	105	6.4	118	80
14/12/2020	41.16	1.73			8.48	2999	2.05	68	80	12	149	92
14/12/2020	44.47	0.27			7.75	3076	5.2	456	80	12	655	77
14/12/2020					8.37	2052	8.77	244	43.7	3.5	31	30
14/12/2020	43.55	0.40			7.47	3355	3.4	772	80	9.8	1150	119
14/12/2020	38.49	9.47	40.96	7.47	7.98	1977	9.09	13	47.7	4.9	40	36
14/12/2020					9.04	3142	1.53	206	80	7.8	400	109
14/12/2020			39.73	14.67	8.45	3702	4.54	150	80	11	218	94
14/12/2020	40.81	2.13			6.38	588	8.03	64	7.3	1.1	139	47
14/12/2020					8.09	3276	0.73	52	2.5	0.5	73	29
14/12/2020	40.79	2.13			6.64	701	5.38	123	8.3	3	241	72
14/12/2020	41.01	1.87	41.90	4.53	7.12	882	3.62	106	19.2	1.8	215	82
14/12/2020	40.37	3.07			6.81	886	1.39	133	17.8	1.9	228	70
15/12/2020					8.24	1655	10.36	28	27.9	3.1	60	20
15/12/2020	39.88	7.73	39.56	19.07	6.64	1790	0.92	1117	26.7	3.3	899	88
15/12/2020					8.58	3674	0.69	1392	80	9.1	1552	223
15/12/2020	37.41	4.93			6.26	2082	0.97	3140	35.5	7.1	2713	827
15/12/2020					8.84	4085	0.43	237	80	5.6	283	79
15/12/2020					8.92	5052	1.35	756	80	14	852	169
15/12/2020	42.79	2.00			9.07	3240	1	151	80	9.3	249	85

15/12/2020			42.58	3.07	8.08	2827	0.67	65	97.5	6.7	242	119
15/12/2020					8.09	3276	0.73	254	80	9.3	337	127
15/12/2020	31.03	189.87	33.43	416.67	7.42	1516	4.56	5	7.5	0.9	NA	30
15/12/2020	34.64	190.67	37.37	46.27	7.24	1787	3.74	26	20.4	1.2	NA	47
15/12/2020			38.76	25.20	8.8	3945	2.33	380	80	15	607	121
15/12/2020					8.61	4053	4.07	439	80	16	678	98
15/12/2020	36.92	38.27	37.29	87.07	7.26	1664	4.12	9	37	4.1	130	44
15/12/2020	35.04	112.67	36.01	116.93	6.96	1653	0.52	71	38.2	4	124	47
15/12/2020	38.22	18.13	39.19	20.27	6.93	965	1.85	338	2.9	0.9	240	86
15/12/2020					7.64	2395	3.26	61	55.4	5.3	133	45
15/12/2020					8.16	3549	7.75	21	80	4.2	NA	47
15/12/2020					7.09	1984	0.38	974	32	11	1273	247
15/12/2020					8.24	1334	10.6	5	5.3	1.6	NA	31
16/12/2020					8.26	586	10.57	32	1.1	1.5	349	256
16/12/2020					7.71	844	8.53	158	11.6	1.8	415	34
16/12/2020			43.40	1.60	8.35	1271	5.46	1328	45.9	6.2	1919	152
16/12/2020	38.71	13.47	39.48	14.27	7.51	1201	8.05	869	17.4	6.2	1580	367
16/12/2020					8.95	1139	9.57	92	38.1	3.2	157	54
16/12/2020	42.61	1.87	44.09	1.07	9.07	1419	7.84	86	44.9	6.7	346	202
16/12/2020					7.8	890	9.94	44	12.3	7.3	173	116
16/12/2020					8.75	2156	7.58	145	66.2	14	456	304
16/12/2020					NA	NA	NA	NA	NA	NA	NA	NA
16/12/2020	36.78	74.13	37.69	35.33	NA	NA	NA	NA	NA	NA	NA	NA
16/12/2020					NA	NA	NA	NA	NA	NA	NA	NA
16/12/2020					NA	NA	NA	NA	NA	NA	NA	NA
16/12/2020					NA	NA	NA	NA	NA	NA	NA	NA
16/12/2020	41.54	5.20			NA	NA	NA	NA	NA	NA	NA	NA
16/12/2020	40.79	3.73			7.58	260	10.24	84	0.8	1.7	168	31
16/12/2020	37.01	35.07	37.93	33.87	7.12	133	10.42	61	4	0.3	122	55
16/12/2020	37.04	35.60	37.36	46.53	7.1	127	10.69	55	4	0.3	144	77

16/12/2020	36.40	51.60	37.11	53.47	9.45	3049	9.26	246	80	11	829	324
16/12/2020	36.62	44.13	36.43	78.27	9.5	3247	9.54	184	80	11	596	329
16/12/2020					7.04	1112	9.42	258	8.9	1	498	107
16/12/2020					7.18	510	8.66	114	3.5	0.8	406	104
16/12/2020					9.27	1548	10.06	71	59.6	9	310	281
16/12/2020	39.65	7.47			9.19	2807	9.02	232	98.3	16	1390	1133
17/12/2020					7.84	1657	10.05	45	9.3	5.2	131	79
17/12/2020	41.02	3.33			7.71	1513	9.73	302	14.6	3	300	19
17/12/2020					NA	NA	NA	NA	NA	NA	NA	NA
17/12/2020					NA	NA	NA	NA	NA	NA	NA	NA
17/12/2020	39.67	15.20	39.80	10.00	NA	NA	NA	NA	NA	NA	NA	NA
17/12/2020	38.04	37.60	39.44	12.27	NA	NA	NA	NA	NA	NA	NA	NA
17/12/2020					9	2207	11.09	626	76.2	6.2	1145	225
17/12/2020					8.21	2344	8.75	1176	37	5.5	1639	481
17/12/2020	41.02	3.33			9.08	2477	10.32	128	94.2	5.7	488	162
17/12/2020	34.75	231.20	36.66	63.47	NA	NA	NA	NA	NA	NA	NA	NA
17/12/2020	33.21	632.93	34.72	212.53	NA	NA	NA	NA	NA	NA	NA	NA
17/12/2020					9.35	2231	6.28	884	109	10	1789	506
17/12/2020	40.75	3.87			9.03	1728	2.35	73	51	4.7	321	167
17/12/2020	40.61	4.27			8.97	2117	3.78	215	49.5	6.9	369	140
17/12/2020					8.84	1168	8.12	262	27.5	5.2	1074	599
17/12/2020	37.48	26.93	37.92	34.13	6.99	634	7.69	79	0.6	1.9	150	55
17/12/2020	39.14	10.00			7.38	2751	9.29	1056	23.2	4.6	1354	219
17/12/2020	39.92	6.27			7.31	2683	5.17	1698	23	4.7	1606	277
17/12/2020					9.61	2170	8.39	417	105	9	842	250
17/12/2020					9.65	2866	12.1	660	111	8.4	1238	270
17/12/2020					9.53	2001	8.51	445	97.8	7.7	1402	265
17/12/2020					9.61	3265	8.65	3118	80	10	1915	516
17/12/2020					8.42	820	10.41	162	2	3.1	333	113
17/12/2020					9.41	2274	11.01	1246	107	17	2241	404
